# Supplementary material for: Direct benefits explain interspecific variation in helping behaviour among cooperatively breeding birds
Source: Nat Commun. 2017 Oct 23;8:1094. doi: 10.1038/s41467-017-01299-5 (PMC5653647; doi:10.1038/s41467-017-01299-5)
Supplement: Supplementary file 1 — Supplementary Information [file 41467_2017_1299_MOESM1_ESM.pdf]

Supplementary Table 1. The effect of shortage of breeding territories (i.e. habitat saturation) on kin discrimination by helpers (the correlation between relatedness and helping) in 21 cooperatively breeding bird species. The effects of average relatedness of helpers and the method to assess kin discrimination (whether the effect of relatedness was assessed on either the *probability* that individuals help or the *amount* of help provided) were corrected for (adjusted r^2 of the model = 0.209). A graphical representation is provided in Fig. 1a.

	estimate	SE	*t*	*P*
intercept	0.525	0.210	2.496	0.023
territory shortage (yes vs no)	-0.381	0.134	-2.841	0.011
average relatedness	-0.056	0.579	-0.098	0.923
method (probability vs amount)	0.004	0.145	0.027	0.979

Supplementary Table 2. Output of Phylogenetic Least Square models to assess the effect of shortage of breeding territories (i.e. habitat saturation) on helping effort (log10 transformed). Table (a) includes all 44 cooperatively breeding species (adjusted r^2 of the model = 0.122), (b) includes 42 species for which helping effort was calculated by excluding non-helping subordinates (adjusted r^2 of the model = 0.216), and (c) includes a subset of 31 species for which the rate of extra-pair paternity (log10 +1 transformed) was known (adjusted r^2 of the model = 0.181). The effects of helper sex ratio, average group size (log10 transformed) and average relatedness of helpers to offspring were corrected for. A graphical representation is provided in Fig. 1b.

**a**. All 44 species	estimate	SE	*t*	*P*
Intercept	1.636	0.214	7.632	<0.0001
territory shortage (yes vs no)	0.172	0.062	2.764	0.009
helper sex ratio	0.042	0.150	0.278	0.782
average group size	-0.016	0.188	-0.085	0.933
average relatedness	0.246	0.234	1.052	0.299

**b**. Excluding non-helping subordinates from helping effort measures (*n* = 42 species)	estimate	SE	*t*	*P*
intercept	1.765	0.219	8.062	<0.0001
territory shortage (yes vs no)	0.206	0.059	3.491	0.001
helper sex ratio	-0.029	0.148	-0.197	0.845
average group size	-0.073	0.181	-0.402	0.690
average relatedness	0.111	0.238	0.465	0.645

**c**. Species with known rates of extra-pair paternity (*n* = 31 species)	estimate	SE	*t*	*P*
intercept	1.445	0.265	5.447	<0.0001
territory shortage (yes vs no)	0.198	0.092	2.141	0.042
helper sex ratio	0.075	0.202	0.368	0.716
log average group size	-0.015	0.233	-0.062	0.951
average relatedness	0.561	0.318	1.767	0.089
rate of extra-pair paternity	0.066	0.065	1.017	0.319

Supplementary Table 3. Output of a Phylogenetic Least Square model, testing the effect of the species-level probability that subordinates inherit the territory on kin discrimination (the correlation between relatedness and helping) in 20 cooperatively breeding bird species. The effects of average relatedness of helpers and the method to assess kin discrimination (whether the effect of relatedness was assessed on either the *probability* that individuals help or the *amount* of help provided) were corrected for (adjusted r^2 of the model = 0.573). A graphical representation is provided in Fig. 2a.

	estimate	SE	*t*	*P*
intercept	0.618	0.162	3.812	0.002
probability of inheritance	-0.013	0.002	-5.046	0.0001
average relatedness	-0.581	0.494	-1.175	0.257
method (probability vs amount)	0.226	0.113	1.992	0.064

Supplementary Table 4. The effect of the probability of territory inheritance on average helping effort. (a) Output of Phylogenetic Least Square models, testing the effect of the species-level probability that subordinates inherit the territory on average helping effort (percentage relative to breeders) in 38 cooperatively breeding bird species (a graphical representation is provided in Fig. 2b). The effects of helper sex ratio, average group size (log10 transformed) and average relatedness of helpers to offspring were corrected for (adjusted r^2 of the model = 0.338). Table (b) provides output from a similar model as (a) but non-helping subordinates were excluded in the calculation of helping effort (adjusted r^2 of the model = 0.376). (c) In a separate analysis, the effect of the rate of extra-pair paternity (log10 +1 transformed) was also corrected for (26 species), which yielded similar results (adjusted r^2 of the model = 0.333). (d) The effect of the probability that subordinates inherit the territory was also similar when only the species inhabiting saturated habitat (i.e. those with shortage of breeding territories) were included (adjusted r^2 of the model = 0.401).

**a**. All species (*n* = 38 species)	estimate	SE	*t*	*P*
intercept	58.169	27.153	2.142	0.04
probability of inheritance	0.741	0.164	4.515	<0.0001
helper sex ratio	-4.222	18.496	-0.228	0.821
average group size	2.271	23.824	0.095	0.925
average relatedness	4.708	30.043	0.157	0.876

**b**. Excluding non-helping subordinates from helping effort measures (*n* = 37 species)	estimate	SE	*t*	*P*
Intercept	70.406	27.651	2.546	0.016
probability of inheritance	0.782	0.169	4.624	<0.0001
helper sex ratio	-14.272	18.829	-0.758	0.454
average group size	3.088	24.176	0.128	0.899
average relatedness	-1.845	30.741	-0.060	0.953

**c**. Species with known rates of extra-pair paternity (*n* = 26)	estimate	SE	*t*	*P*
Intercept	46.321	35.369	1.310	0.205
probability of inheritance	0.759	0.234	3.244	0.004
helper sex ratio	-14.491	25.688	-0.564	0.579
average group size	-4.528	30.017	-0.151	0.882
average relatedness	42.035	45.356	0.927	0.365
rate of extra-pair paternity	10.892	8.415	1.294	0.210

**d**. Only species with territory shortage (*n* = 22)	estimate	SE	*t*	*P*
Intercept	42.373	34.599	1.225	0.237
probability of inheritance	0.836	0.211	3.959	0.001
helper sex ratio	-6.370	25.838	-0.247	0.808
average group size	42.589	37.352	1.140	0.270
average relatedness	-22.367	47.038	-0.476	0.640

Supplementary Table 5. Output of Phylogenetic Least Square that were repeated after conservative exclusion of ambiguous data. The results are the same as for the analyses including all data, for analyses of (a) the effect of the probability that subordinates inherit the territory on kin discrimination (n = 19 species; adjusted r^2 of the model = 0.595; $\lambda < 0.001$), (b) the effect of shortage of breeding territories on helping effort (log10 transformed; n = 40 species; adjusted r^2 of the model = 0.114), and the effect of the probability that subordinates inherit the territory on (c) helping effort (n = 31 species; adjusted r^2 of the model = 0.356). The comparable analyses including all data are provided in Supplementary Tables 1, 3 and 4a, respectively.

**a**. Kin discrimination	estimate	SE	*t*	*P*
intercept	0.547	0.167	3.283	0.005
probability of inheritance	-0.012	0.002	-5.125	0.0001
average relatedness	-0.420	0.497	-0.846	0.411
method (probability vs. amount)	0.235	0.111	2.121	0.051

**b**. Helping effort	estimate	SE	*t*	*P*
Intercept	1.673	0.247	6.780	<0.0001
territory shortage (yes vs no)	0.169	0.064	2.642	0.012
helper sex ratio	0.043	0.173	0.248	0.806
average group size	-0.091	0.200	-0.456	0.651
average relatedness	0.242	0.270	0.894	0.378

**c**. Helping effort	estimate	SE	*t*	*P*
intercept	59.627	29.312	2.034	0.052
probability of inheritance	0.779	0.174	4.478	0.0001
helper sex ratio	0.389	21.547	0.018	0.986
average group size	-2.692	25.503	-0.106	0.917
average relatedness	-3.344	31.899	-0.105	0.917
