# Supplementary material for: Direct benefits explain interspecific variation in helping behaviour among cooperatively breeding birds
Source: Nat Commun. 2017 Oct 23;8:1094. doi: 10.1038/s41467-017-01299-5 (PMC5653647; doi:10.1038/s41467-017-01299-5)
Supplement: Supplementary file 3 — Description of Additional Supplementary Files [file 41467_2017_1299_MOESM3_ESM.pdf]

**File Name**: Supplementary Data 1.

**Description**: A table containing all data used for the comparative study.
